# Detection of long repeat expansions from PCR-free whole-genome sequence data

**DOI:** 10.1101/gr.225672.117

**Published:** 2017-11

**Authors:** Egor Dolzhenko, Joke J.F.A. van Vugt, Richard J. Shaw, Mitchell A. Bekritsky, Marka van Blitterswijk, Giuseppe Narzisi, Subramanian S. Ajay, Vani Rajan, Bryan R. Lajoie, Nathan H. Johnson, Zoya Kingsbury, Sean J. Humphray, Raymond D. Schellevis, William J. Brands, Matt Baker, Rosa Rademakers, Maarten Kooyman, Gijs H.P. Tazelaar, Michael A. van Es, Russell McLaughlin, William Sproviero, Aleksey Shatunov, Ashley Jones, Ahmad Al Khleifat, Alan Pittman, Sarah Morgan, Orla Hardiman, Ammar Al-Chalabi, Chris Shaw, Bradley Smith, Edmund J. Neo, Karen Morrison, Pamela J. Shaw, Catherine Reeves, Lara Winterkorn, Nancy S. Wexler, The US–Venezuela Collaborative Research Group, David E. Housman, Christopher W. Ng, Alina L. Li, Ryan J. Taft, Leonard H. van den Berg, David R. Bentley, Jan H. Veldink, Michael A. Eberle

**Affiliations:** 1 Illumina Incorporated, San Diego, California 92122, USA;; 2 Department of Neurology, Brain Center Rudolf Magnus, University Medical Center Utrecht, Utrecht University, 3584 CX Utrecht, The Netherlands;; 3 Illumina Limited, Chesterford Research Park, Little Chesterford, Nr Saffron Walden, Essex, CB10 1XL, United Kingdom;; 4 Repositive Limited, Future Business Centre, Cambridge CB4 2HY, United Kingdom;; 5 Department of Neuroscience, Mayo Clinic, Jacksonville, Florida 32224, USA;; 6 New York Genome Center, New York, New York 10013, USA;; 7 SURFsara, 1098 XG Amsterdam, The Netherlands;; 8 Academic Unit of Neurology, Trinity College Dublin, Trinity Biomedical Sciences Institute, Dublin 2, Republic of Ireland;; 9 Department of Neurology, Beaumont Hospital, Dublin 9, Republic of Ireland;; 10 Department of Basic and Clinical Neuroscience, Maurice Wohl Clinical Neuroscience Institute, King's College London, London SE5 9RX, United Kingdom;; 11 Department of Molecular Neuroscience, UCL Institute of Neurology, London WC1N 3BG, United Kingdom;; 12 University of Southampton, Southampton SO17 1BJ, United Kingdom;; 13 Sheffield Institute for Translational Neuroscience, University of Sheffield, Sheffield S10 2HQ, United Kingdom;; 14 Columbia University, New York, New York 10032, USA;; 15 Hereditary Disease Foundation, New York, New York 10032, USA;; 16 The US–Venezuela Collaborative Research Group;; 17 Department of Biology, Massachusetts Institute of Technology, Cambridge, Massachusetts 02139, USA

## Abstract

Identifying large expansions of short tandem repeats (STRs), such as those that cause amyotrophic lateral sclerosis (ALS) and fragile X syndrome, is challenging for short-read whole-genome sequencing (WGS) data. A solution to this problem is an important step toward integrating WGS into precision medicine. We developed a software tool called ExpansionHunter that, using PCR-free WGS short-read data, can genotype repeats at the locus of interest, even if the expanded repeat is larger than the read length. We applied our algorithm to WGS data from 3001 ALS patients who have been tested for the presence of the *C9orf72* repeat expansion with repeat-primed PCR (RP-PCR). Compared against this truth data, ExpansionHunter correctly classified all (212/212, 95% CI [0.98, 1.00]) of the expanded samples as either expansions (208) or potential expansions (4). Additionally, 99.9% (2786/2789, 95% CI [0.997, 1.00]) of the wild-type samples were correctly classified as wild type by this method with the remaining three samples identified as possible expansions. We further applied our algorithm to a set of 152 samples in which every sample had one of eight different pathogenic repeat expansions, including those associated with fragile X syndrome, Friedreich's ataxia, and Huntington's disease, and correctly flagged all but one of the known repeat expansions. Thus, ExpansionHunter can be used to accurately detect known pathogenic repeat expansions and provides researchers with a tool that can be used to identify new pathogenic repeat expansions.

Variant callers for small variants such as single-nucleotide polymorphisms and small insertions or deletions typically require multiple reads to completely span the full length of the nonreference allele (DePristo et al. 2011; Raczy et al. 2013). For variants that deviate significantly from the reference, alternative methods such as de novo assembly can be used if the variant is not highly repetitive (Iqbal et al. 2012; Weisenfeld et al. 2014; Li 2015; Chen et al. 2016). Because high-throughput WGS technologies are currently limited to ∼150 base pair (bp) read lengths, variant-calling methods that rely on reads aligned to the reference are subsequently limited to repeat lengths less than 150 bases (Narzisi and Schatz 2015). Many pathogenic repeat expansions have repeats spanning hundreds to thousands of base pairs (Dürr et al. 1996; Gatchel and Zoghbi 2005; Kronquist et al. 2008; Gijselinck et al. 2016), so it has been assumed that short-read sequencing technologies may not be able to identify pathogenic repeat expansions (Loomis et al. 2013; Ashley 2016).

A recently discovered hexamer (GGCCCC) repeat expansion in the *C9orf72* locus is a major cause of both ALS and frontotemporal dementia (DeJesus-Hernandez et al. 2011; Renton et al. 2011; Gijselinck et al. 2012). In particular, the pathogenic repeat length (more than 30 repeats; >180 bp) is present in ∼10% of all ALS patients including ∼40% of familial ALS cases and ∼6%–8% of sporadic ALS cases in some populations (DeJesus-Hernandez et al. 2011; Renton et al. 2011; Gijselinck et al. 2012). The most widely used method to detect *C9orf72* repeat expansions is repeat-primed PCR (RP-PCR) together with fragment length analysis (Akimoto et al. 2014). Interpretation of these PCR results can be challenging due to indels in the flanking regions of the repeat, which can lead to both false positives and false negatives (Akimoto et al. 2014). In addition, these PCR techniques do not provide an estimate of the length of the repeat expansions. Southern blotting is the current gold standard for estimating repeat length, but this method is very challenging to set up; requiring a significant amount of input DNA (generally 10 μg) and suffering from imprecise size estimates due to somatic heterogeneity (Buchman et al. 2013; Akimoto et al. 2014). As WGS is widely adopted for use in precision medicine initiatives (Ashley 2015, 2016; Marx 2015) and large-scale research projects, a reliable method is needed that can identify the presence or absence of potentially pathogenic repeat expansions in WGS data and also determine their approximate length without additional tests.

Here, we present a method to genotype STRs from PCR-free, WGS data implemented in a software package named ExpansionHunter. This method can determine the approximate size of repeats from just a few units in length up to large, pathogenic expansions that may be significantly longer than the read length. To quantify the performance of this algorithm, we first estimate the repeat lengths of two cohorts of ALS patients, all of whom were independently assessed for the presence of the pathogenic *C9orf72* repeat expansion using RP-PCR, and determine the overall sensitivity and specificity of the assay. In addition, we also demonstrate that this method is generally applicable for detecting other repeat expansions by applying it to a set of 152 samples harboring eight other repeat expansions including those that cause fragile X syndrome, Friedreich's ataxia, and Huntington's disease. We also demonstrate the improved accuracy of this method for genotyping STRs shorter than the read length compared to an existing method (lobSTR) on 860 samples for which the size of the longest repeat allele had been experimentally determined. These analyses show that ExpansionHunter is a comprehensive tool for genotyping both short and long repeats. Thus, it can be used to test for the presence of known pathogenic repeat expansions and can be extended as a general STR caller to identify novel pathogenic expansions in population and pedigree studies.

## Results

We performed paired-end, PCR-free, WGS at an average depth of 45× using Illumina HiSeq 2000 (100 bp reads) and Illumina HiSeq X (150 bp reads) systems on two cohorts of patients with ALS (Methods). The first cohort of 2559 patients was used during the development of ExpansionHunter to test the implementation of the algorithm for bugs and to calculate off-target regions (although the core algorithm and its parameters were not informed by these data). The second cohort of 442 patients was used to validate the implementation of the program. All 3001 samples were tested for presence of the *C9orf72* repeat expansion with RP-PCR (Methods). A second RP-PCR test using a different primer set, fragment length analysis, and Southern blotting was performed on 71 samples from the initial cohort, of which 55 had a pathogenic *C9orf72* repeat according to the first RP-PCR (Supplemental Table 2). Additionally, the fluorescent PCR plots were reevaluated for a subset of the samples (Supplemental Fig. 6). As explained in the section on pathogenic *C9orf72* repeat expansion determination, some of the original RP-PCR calls were deemed incorrect based on this reassessment and changed accordingly (Supplemental Table 2), bringing the total counts of expanded and wild-type samples to 212 and 2789, respectively.

**Figure 1.**
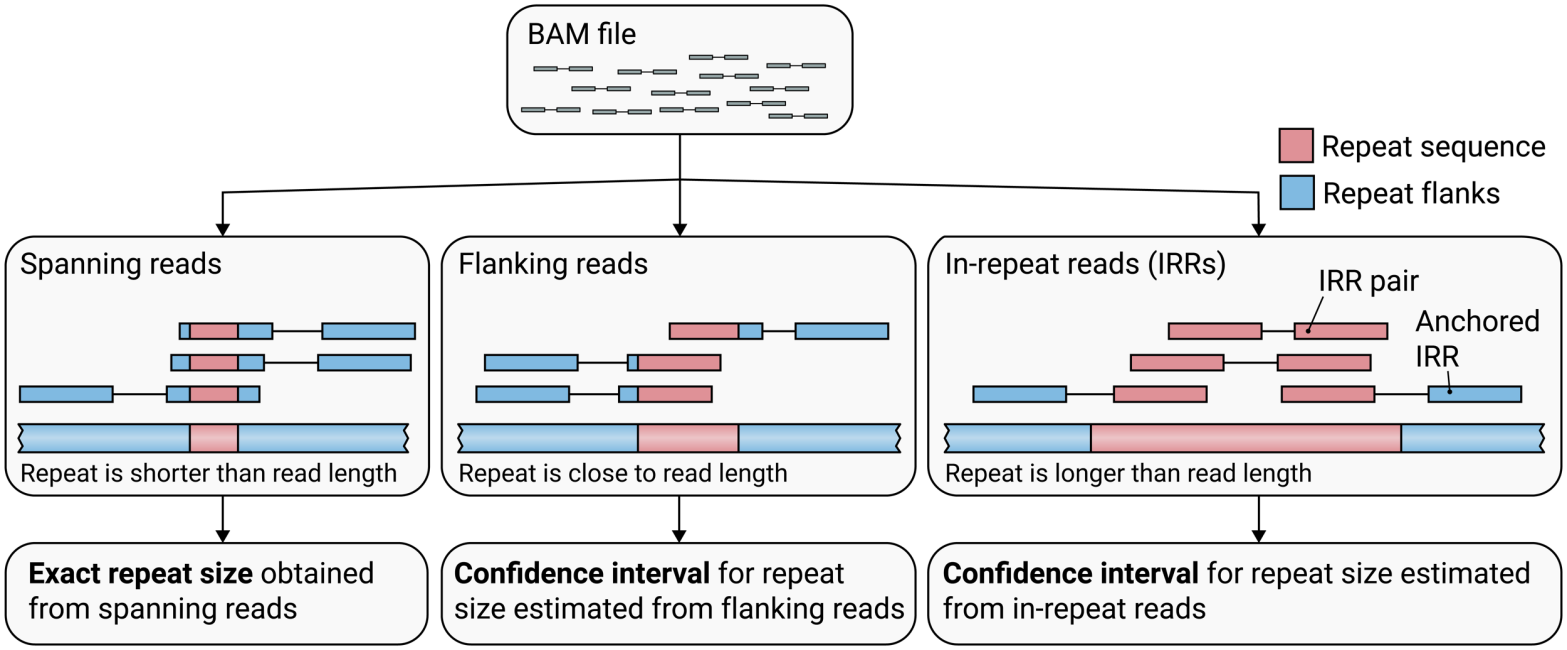
An outline of how ExpansionHunter catalogs reads associated with the repeat locus of interest and estimates repeat lengths starting from a binary alignment/map (BAM) file. (*Left*) Exact sizes of short repeats are identified from spanning reads that completely contain the repeat sequence. (*Middle*) When the repeat length is close to the read length, the size of the repeat is approximated from the flanking reads that partially overlap the repeat and one of the repeat flanks. (*Right*) If the repeat is longer than the read length, its size is estimated from reads completely contained inside the repeat (in-repeat reads). In-repeat reads anchored by their mate to the repeat region are used to estimate the size of the repeat up to the fragment length. When there is no evidence of long repeats with the same repeat motif elsewhere in the genome, pairs of in-repeat reads can also be used to estimate the size of long (greater-than-fragment-length) repeats.

To quantify repeat lengths, we developed an algorithm that identifies reads that either (1) fully span the repeat (spanning reads); or (2) include the repeat and the flanking sequence on one side of the repeat (flanking reads); or (3) are fully contained in the repeat (“in-repeat” reads or IRRs) (Fig. 1). For repeats shorter than the read length of the sequence data we calculate the repeat length using spanning and flanking reads (Fig. 1). To estimate the lengths of repeats that are longer than the read length, we identify and count the IRRs. There are three main hurdles associated with using IRRs to accurately identify repeat expansions that exceed read lengths: (1) identifying IRRs comprised of a potentially error-prone repeat motif; (2) identifying regions in the genome where paired IRRs are systematically (and possibly incorrectly) placed by the aligner; and (3) estimating the repeat length based on the total number of IRRs identified. Here, we describe how we solve these problems to identify and characterize expanded repeats accurately.

### On-target IRRs

Identifying reads originating in highly repetitive regions can be difficult because sequencing error rates are higher in low complexity regions such as homopolymers and STRs (Benjamini and Speed 2012), so we implemented a weighted measure that penalizes base mismatches at low-quality bases less than mismatches at high-quality bases (Methods). To identify IRRs that originate within the *C9orf72* repeat, we extracted all read pairs in which one read is an IRR and the other read aligns with high accuracy (mapping quality [MAPQ] at least 60) within 1 kbp of the *C9orf72* repeat locus. We call such reads anchored IRRs. Because the mates of anchored IRRs align uniquely near the target repeat, we are confident that the IRRs come from the *C9orf72* repeat locus. Anchored IRRs can be used to estimate the size of repeats that are longer than the read length but shorter than the fragment length. For repeats exceeding the fragment length, the number of anchored IRRs provides a lower bound for the repeat length.

### Off-target IRRs

The library preparation used for these sequencing experiments had a mean fragment size of ∼350–450 bp, but the *C9orf72* repeat expansion can be >10 kbp in length (Gijselinck et al. 2016). This means that in addition to anchored IRRs, pairs in which both mates are IRRs could be present in samples with the *C9orf72* repeat expansion (Fig. 1). Because the expanded repeat is not present in the reference, these paired IRRs may not align to the *C9orf72* repeat locus and could either not align at all or misalign to a different locus in the genome (Church et al. 2015; Gijselinck et al. 2016). To identify unaligned or misaligned IRRs, we tested every (MAPQ = 0) read in all 182 expanded ALS samples of the first cohort identified by the first round of RP-PCR as having the *C9orf72* repeat expansion. These 182 samples contained 29,619 poorly mapped paired IRRs altogether—33% of these were unaligned and 67% resided in 29 loci (which we term off-target regions), and only 0.1% were located elsewhere (Methods). Conversely, when we performed the same analysis on 182 random samples without the *C9orf72* repeat expansion according to RP-PCR, we did not find paired IRRs in any genomic locus.

**Figure 2.**
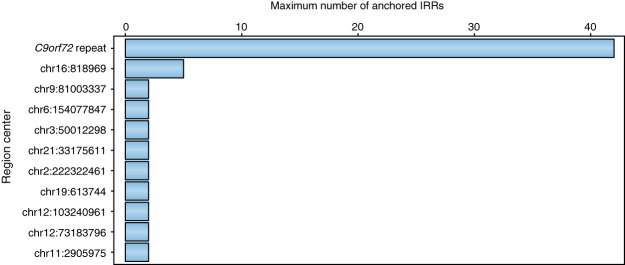
The maximum number of anchored IRRs observed in any of the 2559 samples from cohort one for the genomic loci with at least two anchored IRRs in at least one sample (Methods).

We next analyzed positions where the mates of anchored IRRs aligned in all 2559 samples from cohort one. For each sample, we identified all the anchored IRRs and then grouped the IRRs anchored within 500 bp of one another. The *C9orf72* repeat locus had many anchored IRRs in nearly all samples with a pathogenic repeat expansion (178 samples had five or more anchored IRRs and 160 had 10 or more) indicating that the repeat exceeds the read length in these samples as expected. Only 10 genomic loci had more than one IRR anchored outside of the *C9orf72* repeat locus in any of these samples (Fig. 2). Based on this, we considered all paired IRRs to originate from the *C9orf72* repeat locus and included them in the size estimation when testing this repeat.

### Repeat size estimation

Improvements to short-read sequencing technology such as PCR-free sample preparation minimize the GC bias that previously bedeviled PCR-based WGS data (Meienberg et al. 2016). This is illustrated by the improved coverage of high GC regions such as the *FMR1* repeat (Supplemental Fig. 5). These improvements enabled us to estimate the length of a region by the number of reads that originate from it even for regions with high GC content. By assuming that the number of reads that originate in a given region follows a binomial distribution, we were able to estimate the size of the repeat by the number of IRRs. The number of IRRs in individual samples ranged from 0 to 1314 corresponding to estimated *C9orf72* repeat sizes of up to 7152 bp.

For shorter alleles, the sizes of repeats were determined using spanning reads (Fig. 1). For repeats that are close to the read length, the repeat may be too long to produce spanning reads but too short to produce IRRs. Therefore, the algorithm also uses flanking reads (Fig. 1) to estimate the repeat size (Methods). In the 2559 samples of cohort one, 1.6% (40) of the samples had a repeat size estimated using only flanking reads that resulted in repeat size estimates from 18 to 144 bp (Supplemental Table 2).

ExpansionHunter computes the maximum-likelihood genotype consisting of candidate repeat alleles determined by spanning, flanking, and in-repeat reads (Methods). When both alleles are longer than the read length, the algorithm computes intervals for possible sizes of short and long repeats based on the two extreme cases: (1) All reads come from one haplotype; or (2) half the reads come from each haplotype.

### Pathogenic *C9orf72* repeat expansion detection

The *C9orf72* repeat sizes for both ALS cohorts determined by our method were compared to the RP-PCR results (Supplemental Table 2). Cases in which the estimated confidence interval for repeat size overlapped the pathogenic *C9orf72* repeat size cutoff (i.e., the lower bound was less than 30 repeats and the upper bound was greater than 30 repeats) were defined as “gray” and considered “long” in all sensitivity/specificity calculations. Using the original RP-PCR calls as the ground truth, we identified 11 discrepant calls between our method and RP-PCR, resulting in the overall sensitivity and specificity of 98.6% and 99.6%, respectively, for the WGS-based calls. Because RP-PCR involves many manual steps and, as has been shown previously, could be prone to error (Akimoto et al. 2014), we performed an additional analysis of the discrepant calls to understand the source of these conflicts.

Of the 11 samples with a discrepant classification between our method and the RP-PCR calls, eight were “EH positive/RP-PCR negative” (positive = expansion; negative = normal); however, each of these discrepant calls had at least 13 anchored IRRs, which constitutes strong supporting evidence for a pathogenic repeat expansion in these samples (Supplemental Table 4; Supplemental Fig. 6). Predicting the repeat length using only the anchored reads also supported the pathogenic repeat expansion sizing in all eight “EH positive/RP-PCR negative” samples. Conversely, two of the three “EH negative/RP-PCR positive” samples had compelling read-level evidence supporting their negative status: The read-level data supported repeat alleles of two distinct sizes, each spanning fewer than 30 repeat units. Specifically, one sample contained 10 spanning reads with a repeat of size 2 and 10 spanning reads with a repeat of size 5, whereas the other sample had a size estimate just under the pathogenic cutoff (16 to 26 repeat units). The final “EH negative/RP-PCR positive” sample had just one allele identified (consisting of two repeat units), but the number of spanning reads (38) was consistent with the read depth (mean depth = 44×) in this sample supporting a homozygous, nonpathogenic variant (Supplemental Table 4; Supplemental Fig. 6).

We also reevaluated the original RP-PCR calls for eight samples (Supplemental Fig. 6) and performed an additional RP-PCR and fragment length analysis for 71 samples (Supplemental Table 2). This analysis showed that in 10 of the 11 conflicting calls, the original RP-PCR call was incorrect; therefore, ExpansionHunter and the RP-PCR results were consistent (Supplemental Table 4). The remaining conflict was also resolved after an additional RP-PCR was performed on this sample with different primers (Methods). Additionally, one sample classified as gray range by ExpansionHunter was reclassified from RP-PCR positive to RP-PCR negative. Fragment length analysis estimated the repeat in this sample to be between 28 and 30 repeat units. Because we count gray range samples as expanded, we now consider this sample as misclassified by our analysis, although the experimental size range overlaps the size range predicted by ExpansionHunter. The remaining 67 samples had no conflicts between ExpansionHunter and RP-PCR in either the first or second RP-PCR analysis. Reclassifying the original calls based on this additional analysis, the total number RP-PCR expanded samples increased from 208 to 212.

Comparing our calls against the updated RP-PCR results showed that the only discrepancies in classification are due to the seven “gray” calls, in which the samples likely have repeat lengths close to 30 repeat units (Table 1). Because we consider “gray” calls as expanded, this method produced just three false positives (EH gray/RP-PCR negative) and no false negatives. Overall, ExpansionHunter correctly flagged all (212/212, 95% CI [98.7%, 100%]) of the expanded samples as either expansions (208) or potential expansions (4). Additionally, 99.9% (2,786/2,789, 95% CI [99.7%, 100%]) of the nonexpanded samples were correctly classified, and the three discrepant calls were labeled as “gray” by ExpansionHunter.

**Table 1.**
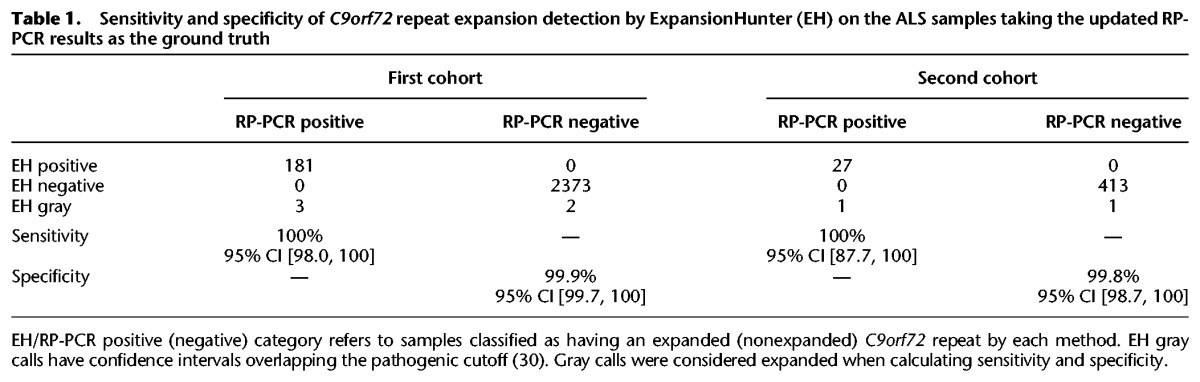
Sensitivity and specificity of *C9orf72* repeat expansion detection by ExpansionHunter (EH) on the ALS samples taking the updated RP-PCR results as the ground truth

### Repeats shorter than the read length

To quantify the accuracy of our method for alleles shorter than the read length, we compared our results to those obtained on 860 samples for which the size of the longest allele was estimated using fragment length analysis (Supplemental Table 2). In addition, we also analyzed these samples using the STR calling tool lobSTR (Gymrek et al. 2012). It should be noted that lobSTR is designed for general genome-wide STR calling based on spanning reads and is limited to calling repeat lengths shorter than the read length, so it may not make a call for longer repeats. In this comparison, the ExpansionHunter calls agreed with the fragment length analysis in 821 (95.5%) of the samples, and the lobSTR calls agreed with the fragment length analysis in 734 (85.3%) of the samples. Of the 39 ExpansionHunter repeat sizes that did not agree with the fragment length analysis, 20 (51%) were in agreement with the lobSTR calls, and the remaining 19 calls were predicted to be longer repeats (spanning eight or more repeat units) where lobSTR is less likely to make a call (Supplemental Tables 2, 5).

**Figure 3.**
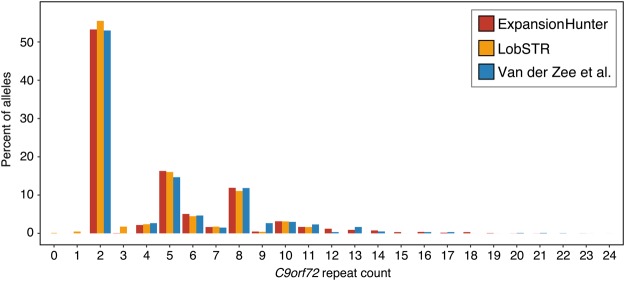
Distribution of ExpansionHunter and lobSTR allele sizes of the *C9orf72* repeat in the 1770 samples with 150 bp reads from cohorts one and two, compared with those of the FTLD cohort of 318 samples from a previous study (van der Zee et al. 2013).

Next, we analyzed the 1770 samples that were sequenced with 2×150 bp reads to get the distribution of the repeat lengths identified from spanning reads in the *C9orf72* repeat. The distribution determined by this analysis is very similar to the results obtained in a previous study (van der Zee et al. 2013) that used an alternative repeat-primed PCR assay and a short tandem repeat (STR) fragment length assay with flanking primers optimized for alleles with high GC content (STR-PCR) allowing exact sizing of normal lengths (Fig. 3). This indicates that we can accurately resolve the length of the short repeats. Because of the requirement for reads to span the STR fully, the maximum repeat size called by lobSTR is 11 repeats, although 4.2% (145 of 3394) of our alleles are sized greater than 11 repeats.

### Applying ExpansionHunter to other repeat expansions

In addition to the *C9orf72* repeat, several other pathogenic repeat expansions have been identified (McMurray 2010). To demonstrate the general applicability of our method, we tested eight other pathogenic repeat loci by sequencing and genotyping 152 samples with known expansions and 26 controls. The sample set contains 98 Coriell samples (https://catalog.coriell.org) from 64 families with a variety of repeat expansions associated with dentatorubral-pallidoluysian atrophy (DRPLA, *ATN1* gene), fragile X Syndrome (FXS, *FMR1* gene), Friedreich's ataxia (FRDA, *FXN* gene), Huntington's disease (HD, *HTT* gene), myotonic dystrophy type 1 (DM1, *DMPK* gene), spinocerebellar ataxia type 1 (SCA1, *ATXN1* gene), spinocerebellar ataxia type 3 (SCA3, *ATXN3* gene), and spinal and bulbar muscular atrophy (SBMA, *AR* gene). In addition to the Coriell samples, our data include 54 samples with *HTT* expansions obtained from a rural fishing village in Venezuela with the highest concentration of Huntington's disease in the world (Wexler et al. 2004). These 54 samples were processed with a different alignment software (Li and Durbin 2009) allowing us to demonstrate that ExpansionHunter is compatible with other commonly used short-read aligners.

Taken together, these 152 samples represent different repeats with a variety of repeat sizes including normal, premutated, and fully expanded repeats. Premutated repeats are nonpathogenic repeats that are predisposed to become pathogenic/fully expanded in subsequent generations. Normal/premutation transitions for the repeats that we target ranged between 87 and 165 bases and premutation/full expansion transitions ranged between 114 and 600 bases. The repeats in the *HTT*, *ATXN*1, and *AR* genes are short enough that anchored IRRs alone are sufficient to detect the expansion. For the expansion in the *FMR1* gene, we included off-target reads using the methodology we developed for the *C9orf72* repeat to improve our ability to quantify large repeats. We did not include off-target locations for the other, potentially long repeats because the corresponding motifs (CAG and AAG) are common enough that we could not resolve which repeat the paired IRRs originated from.

**Figure 4.**
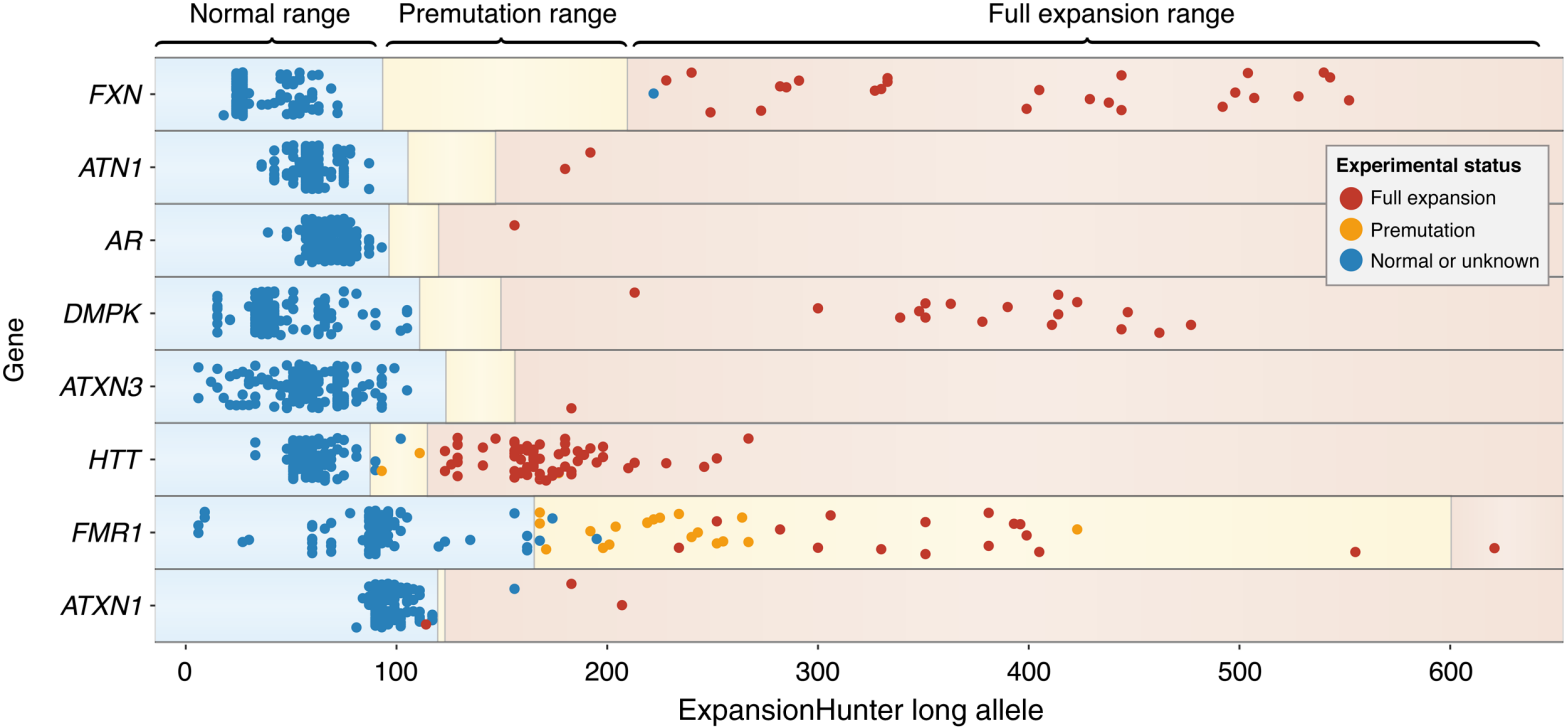
Sizes of the longer repeat alleles predicted by ExpansionHunter in the 152 samples identified as having either a premutation or an expansion at loci associated with eight different diseases and 24 additional control samples. Circles indicate the most-likely repeat length of the longer allele in base pairs for a sample identified with a premutation (orange) or expansion (red), and the blue circles show the predicted repeat lengths for the controls. The controls include samples with measurements showing that they fall in the “normal” range and samples that have a different repeat expansion. Thus, each sample will have one circle for each of the eight repeat expansions. The regions are shaded to indicate the normal ranges (blue), premutation ranges (yellow), and expansion sizes (light red) (McMurray 2010). Additional information is available in Supplemental Tables 7 and 8.

Figure 4 depicts the sizes of the longer repeat allele determined by ExpansionHunter. Each of the 152 samples was tested for eight repeat expansions, one of which is expected to be expanded and the rest wild type. All 24 control samples were similarly tested across all eight expansions. Our method identified all repeats expected to be premutated (orange circles) or fully expanded (red circles). The categorization was correct for all repeats with an exception of the *FMR1* repeats, in which 15 of 16 repeats were estimated by ExpansionHunter to be premutations instead of full expansions, and one *ATXN1* expanded sample was identified in the normal range.

Although we correctly identified all but one of the expansions, there was one “control” sample showing the *FXN* expansion and three “control” samples with the *FMR1* repeat size at the low end of the premutation range. Both of these results are unsurprising due to the higher carrier frequencies for these two repeats: The carrier frequency is 1:90 for *FXN* (Zamba-Papanicolaou et al. 2009) and 1:178 for the *FMR1* premutation (Hantash et al. 2011). Additionally, there was one “control” sample showing an *ATXN1* expansion. The final three putative FP samples were identified in the *HTT* repeat and include a mother and son who were both sized at 30 repeats (bottom of the premutation range), and a third sample with 34 repeats, which is small enough for an individual to be unaffected. Visual inspection of the reads supported the ExpansionHunter calls in these samples.

Although ExpansionHunter is intended for unbiased (e.g., PCR-free) sequence data, 12 of the samples studied here were sequenced with a PCR step in the sample preparation. These comprised nine samples with either a premutation or expansion at the *HTT* gene and three controls. These samples were correctly classified for the *HTT* repeat despite the high GC content of this CAG repeat (67%). Conversely, in these samples the *FMR1* repeat length could not be assessed for all but one of these samples: Four samples had no reads covering the repeat and seven were covered very poorly (one to three reads covering the repeat) and produced excessively small repeat lengths. For example, these seven samples were all sized at fewer than 10 repeats, whereas for the other 157 samples sequenced without PCR, only four samples had alleles shorter than 20 repeats and the smallest of these spanned 14 repeats.

## Discussion

We developed a software tool that can identify pathogenic repeat expansions from paired-end, PCR-free WGS data. Comparing against the results obtained with a widely used wet lab protocol for identifying pathogenic repeat expansions in the *C9orf72* locus, ExpansionHunter was able to correctly classify all expanded samples as either expansions (208) or potential expansions (4) and 2786 of the 2789 wild-type samples. Some samples are classified as potential expansions, because there is an uncertainty associated with repeats longer than the read length. In a clinical setting, such calls would trigger a follow-up analysis; so all the expansions were flagged in this analysis.

We also demonstrated that our method generalizes to other repeats by correctly identifying the validated repeats from 152 samples with eight other pathogenic repeat expansions. In total, we examined five repeat motifs (CTG, GAA, CGG, CAG, and GGCCCC) at nine different genomic locations and demonstrated that ExpansionHunter can detect repeat expansions in a variety of sequence contexts. It is particularly important that our method works on the very high (100%) GC repeats in *FMR1* (CGG) and *C9orf72* (GGCCCC) genes, where both coverage biases and error rates may be elevated. Comparing our size estimates with Southern blot experiments indicated that our method may underestimate sizes of some very long repeats, particularly those in the *FMR1* and *AR* genes (Fig. 4; Supplemental Table 7). This discrepancy could be due to mosaicism of expanded *FMR1* repeats (in fact, several of the samples with *FMR1* expansions were identified as mosaic in the Coriell database). In addition to mosaicism, other factors such as higher error rates and GC biases may play a role in causing this method to underestimate the size of these long repeats. Still, the *FMR1* expansions were generally sized as being longer compared to the *FMR1* premutations, indicating that it may be possible to calibrate the size estimates and account for errors not related to mosaicism. Future work will concentrate on quantifying this behavior and improving the size estimates for these long repeats.

ExpansionHunter is designed for PCR-free WGS data, and comprehensive detection of large pathogenic repeats is unlikely to work with whole-exome sequence data because (1) many repeats of interest are not exonic; and (2) size estimates for large repeats require assumptions about the average number of reads per base. Additionally, some important repeats, like the repeat in *FMR1* gene that causes fragile X syndrome, are 100% GC and are underrepresented in sequence data that includes a PCR step during sample preparation.

If there is no evidence of long repeats with the same repeat motif elsewhere in the genome (e.g., the GGCCCC repeat in *C9orf72*), both anchored and paired IRRs can be utilized to estimate the full length of the repeat. If, on the other hand, multiple long repeats with the same repeat motif exist, then the size of the repeat is estimated only from anchored in-repeat reads and so is capped by the fragment length. Although the off-target reads improve the size estimates produced by this method, most repeats are classified as pathogenic much closer to the read length (e.g., Fig. 4). Because of this, in most cases the anchored reads are sufficient to identify pathogenic repeats and, for example, all 212 of the *C9orf72* expanded samples were also identified even when using only the anchored IRRs to estimate the repeat length.

A major benefit of our tool is that it enables researchers to screen for all known repeat expansions using a single whole-genome sequencing run. As the throughput of WGS increases and the cost decreases, WGS may soon become the basis for frontline tests for repeat expansions and other genetic disorders. Theoretically, long reads can also identify many of the longer repeat expansions (Loomis et al. 2013), but those technologies are still too expensive to be routinely used for whole-genome screening. At the same time, because the substitution and indel error rates in these long reads range from 10% to 30% (Sović et al. 2016; Bao and Lan 2017), it may be difficult to classify the repeat confidently when its size is close to the normal-premutation or premutation-expansion boundary cutoffs unless the samples are sequenced to high depth.

Because repeat expansions may expand from generation to generation, pathogenic repeats may show little or no linkage disequilibrium with the surrounding variants. Thus, association studies based solely on SNPs may be blind to these highly polymorphic risk alleles. An important result highlighted in Figures 3 and 4 is that ExpansionHunter is able to size both short and long repeats. This will allow researchers to quantify repeat lengths of all STRs genome-wide and discover novel pathogenic repeat expansions agnostically. Although this method can be used to quantify every repeat in the genome, ExpansionHunter is a targeted tool that requires an STR to be specified by its reference coordinates and repeat motif. Additional work is ongoing to develop a genome-wide STR database that ExpansionHunter can use to target any STR genome-wide. As association studies based on high-depth WGS data become more widespread, it will be possible to discover new, previously undetected repeat expansions by genotyping them across the population with ExpansionHunter.

## Methods

### Whole-genome sequencing

Whole-genome shotgun sequencing was performed for all of the samples analyzed in this study. For 1231 of the ALS samples, we used TruSeq DNA PCR-free sample preparation with 100 bp paired-end reads sequenced on Illumina HiSeq 2000 instruments. The remaining 1770 ALS samples, the 128 Coriell samples, and 42 of the Huntington samples used TruSeq DNA PCR-free sample preparation with 150 bp paired-end reads sequenced on Illumina HiSeq X instruments. The final eight samples used TruSeq Nano library preparation with 150 bp paired reads sequenced on Illumina HiSeq X instruments.

### *C9orf72* PCR

Repeat-primed PCR (RP-PCR) was performed on 50–300 ng gDNA with 1× FastStart Mix (Roche), 0.9 M Betaine, 5% DMSO, 1 mM MgCl_2_, 0.2 mM 7-deaza-dGTP, 0.6–1.3 µM F-primer ([6FAM]AGTCGCTAGAGGCGAAA(GC)), 0.3–1.3 µM R-primer (TACG CATCCCAGTTTGAGACGGGGGCCGGGGCCGGGGCC(GGGG)), 0.6–1.3 µM anchor-primer (TACGCATCCCAGTTTGAGACG) in a total volume of 16–30 µL, with this protocol: 15 min 95°C; 2 cycles 1 min 94°C, 1 min 70°C, 3 min 72°C; 3 cycles 1 min 94°C, 1 min 68°C, 3 min 72°C; 4 cycles 1 min 94°C, 1 min 66°C, 3 min 72°C; 5 cycles 1 min 94°C, 1 min 64°C, 3 min 72°C; 6 cycles 1 min 94°C, 1 min 62°C, 3 min 72°C; 7 cycles 1 min 94°C, 1 min 60°C, 3 min 72°C; 8 cycles 1 min 94°C, 1 min 58°C, 3 min 72°C; 5 cycles 1 min 94°C, 1 min 56°C, 3 min 72°C; 10 min 72°C. The PCR product was analyzed on an ABI 3730 DNA Analyzer (Applied Biosystems) with PeakScanner software (v1.0). A characteristic stutter pattern was considered evidence of a *C9orf72* repeat expansion. Fluorescent PCR was performed as previously described (DeJesus-Hernandez et al. 2011).

### Confirmation of *C9orf72* RP-PCR results

The presence of a repeat expansion was determined in a blinded fashion using a two-step PCR protocol (DeJesus-Hernandez et al. 2011). Genomic DNA was PCR amplified with genotyping primers and one fluorescently labeled primer, followed by fragment length analysis with an ABI 3730 DNA analyzer and GeneMapper software (v5). A single PCR fragment could either indicate a homozygous variant or a pathogenic repeat expansion. Subjects with a single PCR fragment were selected for RP-PCR, and PCR products were analyzed with an ABI 3730 DNA Analyzer and GeneMapper software. If the RP-PCR revealed a characteristic stutter pattern, these individuals were screened using Southern blotting techniques, as described previously (DeJesus-Hernandez et al. 2011). A total of 7–10 µg of genomic DNA was digested with XbaI (Promega) and electrophoresed in a 0.8% agarose gel. DNA was then transferred to a positively charged nylon membrane (Roche), cross-linked, and subsequently hybridized with a digoxigenin (DIG)-labeled probe. Expansions were visualized with anti-DIG antibody (Roche) and CDP-Star substrate (Roche) on X-ray film.

### Identifying IRRs

To test if a read fully consists of the repeat motif, we compared it to the perfect repeat sequence that was the closest match under the shift and reverse complement operations (e.g., a read originating in a CAG repeat can consist of repetitions of either CAG, AGC, GCA in the forward orientation or CTG, TGC, GCT in the reverse orientation). To do the comparison, we defined the weighted purity (WP) score metric that assigns each matching base a score of 1, each low-quality mismatch a score of 0.5 and each high-quality mismatch a score of −1. After normalization of the sum of per-base scores for the total read length, the WP ranges from −1 to 1. We defined IRRs as reads that achieve WP of 0.9 or higher (Supplemental Methods).

### Identifying off-target regions

Paired IRRs originating from expanded STRs may align to other genomic locations, especially if the STR is short in the reference genome at the target location. We refer to the loci where IRRs may misalign as off-target regions. Identifying off-target regions enables us to reduce the search for IRRs to a few regions instead of the whole genome. In order to obtain off-target regions for the *C9orf72* repeat, we searched through the 182 samples in cohort one that had an expanded repeat according to the original RP-PCR results to identify all the GGGGCC IRRs. The search was performed through the whole genome for read pairs with a low mapping quality (MAPQ = 0) and a weighted purity score of at least 0.9. The mapping positions of all identified IRRs were merged if they were closer than 500 bp, and the resulting 29 loci that were present in five or more samples were designated as off-target regions (Supplemental Fig. 4) and were used to find additional reads from the *C9orf72* repeat expansion.

### Repeat size estimation from IRRs

We assume that the probability of observing a read starting at a given base follows the Bernoulli distribution with the probability of success parameter, π, equal to the ratio of the read depth to the read length. Thus, starting positions of the reads occurring in a given region define a Bernoulli process, and the number of reads starting in the region follows a binomial distribution. If *r* is the read length, then one of the terminal bases of any IRR must start at least *N* − *r* bases away from the flanks of the repeat. The probability of observing *i* such reads is

P(i,N−r)=(N−ri)πi(1−π)N−r−i.

Because we have the estimates for *i* (the number of IRRs) and π (the probability that there is a read starting at a given base), *N* (the repeat size) can be estimated by *r* + *i*/π. The confidence interval for the repeat size is estimated by the parametric bootstrap method (Rice 2007). The same procedure is used to obtain point estimates and confidence intervals for repeat sizes from flanking reads. The confidence interval is truncated according to the size of the longest repeat sequence observed in a flanking read.

### Repeat size determination from spanning reads

The reads spanning the repeat are identified from all the reads that aligned within 1 kb of the target repeat region. Each of these reads is tested for the presence of the repeat motif, after which the flanking sequences of the repeat in the read is aligned to the flanking sequences of the repeat in the reference. To be considered spanning, a read must achieve a WP score of 0.9 across the repeat sequence and its flanks. Furthermore, the flanking sequence must have at most two fewer high-quality mismatches or four fewer low-quality mismatches compared to the sequence obtained by extending the repeat. So, if the flanking sequence is similar to the repeat motif, then more flanking sequence is required to identify the end of the repeat and the beginning of the flanking sequence.

### Repeat genotyping

Genotype probabilities for repeats of size up to the read length are calculated using a similar model as the one used for SNPs (Li et al. 2009). Namely, *P*(*G*|*R*) = *P*(*R*|*G*) · *P*(*G*)/*P*(*R*) where the genotype G is a tuple of repeat sizes with the number of entries equal to the ploidy of the chromosome containing the repeat. The probability *P*(*R*|*G*) is expressed in terms of the probabilities *P*(*r*_*i*_|*H*_*i*_) for individual reads *r*_*i*_ and repeat alleles *H*_*i*_ as described (Li et al. 2009).

If *r*_*i*_ is a spanning read containing *m* repeat units, *P*(*r*_*i*_|*H*_*i*_ = *n*) = π · *f*(*m*| *p*, *n*, *s*), where π is defined as above (“Repeat size estimation from IRRs”). The frequency function *f* is defined by *f*(*m*|*p*, *n*, *s*) ∼ *p*(1 − *p*)^*d*^, where *m*, *n*, *s* are non-negative integers bounded by the maximum number of repeat units in a read which we denote by *u*, *p* ∈ (0, 1) corresponds to the proportion of molecules with repeat of the expected size, and *d* = |*n* − *m*| if |*n* − *m*| < *s* and *d* = *s* otherwise. Note that *f* is defined similarly to the geometric frequency function with parameter *d* representing the deviation from *n*, the expected repeat size (which can be at most *s*). If *r*_*i*_ is a flanking or in-repeat read containing *m* repeat units, $P(r_{i}|H_{i}=n)=\pi \cdot \overset{u}{\underset{i=m}{\sum }}f\;(i|p,n,s)$. In all our analyses, the parameters *p* and *s* were set to 0.97 and 5. The values were chosen to maximize Mendelian consistency of genotype calls in Platinum Genome pedigree samples (Eberle et al. 2017) on an unrelated set of repeats.

We use read-length-sized repeats as a stand-in for repeats longer than the read length. If only one allele is expanded, we estimate the full size of the repeat as described above. If both alleles are expanded, the size intervals are estimated similarly by assuming that between 0 and 50% of in-repeat reads come from the short allele and between 50% and 100% of in-repeat reads come from the long allele.

### Software availability

ExpansionHunter is written in C++, and its source code is included in the Supplemental Materials. The software is licensed under GPLv3.0, and the binaries, source code, and documentation are also freely available at https://github.com/Illumina/Expansion Hunter.

## Data access

WGS for Coriell samples, WGS reads from *C9orf72* repeat region for Project MinE samples, and *HTT* repeat region for Huntington's disease samples from this study have been submitted to the European Genome-phenome Archive (EGA; https://www.ebi.ac.uk/ega/home) under accession numbers EGAS00001002462, EGAS00001002598, and EGAS00001002593, respectively. The following cell lines/DNA samples were obtained from the NIGMS Human Genetic Cell Repository at the Coriell Institute for Medical Research: NA04724, NA05446, NA05539, NA05676, NA06477, NA06591, NA05470, NA05438, NA06075, NA04567, NA05164, NA04648, NA05152, NA23378, NA23374, NA23300, NA03986, NA03989, NA03990, NA03696, NA03759, NA04034, NA03697, NA03132, NA03756, NA13716, NA13717, NA03816, NA04079, NA14519, NA15850, NA15847, NA15848, NA16197, NA16200, NA16202, NA16203, NA16205, NA16209, NA16210, NA16212, NA16216, NA16213, NA16215, NA16214, NA16227, NA16229, NA16228, NA16237, NA16243, NA16240, NA16207, NA06895, NA04025, NA04926, NA05131, NA05185, NA09145, NA09237, NA07063, NA07539, NA06890, NA06905, NA07536, NA07540, NA07542, NA06910, NA06894, NA07541, NA07175, NA06889, NA06893, NA06896, NA07538, NA07537, NA06897, NA07174, NA06903, NA07543, NA06852, NA06891, NA06907, NA06906, NA06892, NA06904, NA06968, NA07294, NA09316, NA09317, NA09497, NA07730, CD00014, NA03200, NA20235, NA20238, NA20237, NA20239, NA20242, NA20243, NA20230, NA20232, NA20233, NA20234, NA20236, NA20231, NA20240, NA20241, NA20244, NA07862, NA13509, NA13515, CD00022, NA13507, NA13508, NA13510, NA13511, NA13512, NA13513, NA13514, NA13503, NA13504, NA13505, NA13506, NA06926, NA13536, NA13537, NA06151, NA23709.

## Competing interest statement

Some of the authors are employees of Illumina, Inc.

## Supplementary Material

Supplemental Material
