# Supplementary material for: Detection of long repeat expansions from PCR-free whole-genome sequence data
Source: Genome Res. 2017 Nov;27(11):1895–903. doi: 10.1101/gr.225672.117 (PMC5668946; doi:10.1101/gr.225672.117)
Supplement: Supplemental Material [file supp_27_11_1895__index.html]

Detection of long repeat expansions from PCR-free whole-genome sequence data — Supplemental Material 

# Detection of long repeat expansions from PCR-free whole-genome sequence data

## Supplemental Material

- Supplemental\_Information.docx
- Supplemental\_SourceCode\_ExpansionHunterv2.5.3.zip
- Supplemental\_Table\_2.xlsx
- Supplemental\_Table\_4.xlsx
- Supplemental\_Table\_5.xlsx
- Supplemental\_Table\_6.xlsx
- Supplemental\_Table\_7.xlsx
- Supplemental\_Table\_8.xlsx
- Supplemental\_Fig\_6.pdf
