# Supplementary material for: Detection of long repeat expansions from PCR-free whole-genome sequence data
Source: Genome Res. 2017 Nov;27(11):1895–903. doi: 10.1101/gr.225672.117 (PMC5668946; doi:10.1101/gr.225672.117)
Supplement: Supplemental Material [file supp_gr.225672.117_Supplemental_Information.docx]

Supplementary Information

## 1 Supplementary Tables and Figures

|  | Sequencing platform | # Samples | RP-PCR *C9orf72* expanded (%) |
| --- | --- | --- | --- |
| Cohort 1 | HiSeq 2000 | 1,231 | 75 (6.1) |
|  | HiSeq X | 1,328 | 107 (8.1) |
| Cohort 2 | HiSeq X | 442 | 26 (5.9) |

**Supplementary Table 1.** Sequencing platform, number of samples and the number/percentage of samples with pathogenic *C9orf72* repeat expansions identified by the initial RP-PCR analysis for each cohort.

**Supplementary Table 2.** Summary information about each of the 3,001 ALS patient samples: sample identifier, cohort, WGS read length, the *C9orf72* RP-PCR call/fragment length analysis results (original, 2^nd^ and final calls), the Southern blot repeat size and position, read depth, repeat genotype predicted by lobSTR and ExpansionHunter. Samples sequenced with HiSeq 2000 have 100bp read lengths and samples sequenced with HiSeq X have 150bp read lengths. The PCR wild type (‘wt’) samples were estimated by RP-PCR to have a *C9orf72* repeat size smaller than 30 repeats, while ‘long’ samples have a repeat size of 30 or more. For 860 samples the repeat size of the longest ‘wt’ allele was estimated using fragment length analysis. The Southern blot positions correspond to the results shown in Supplementary Figure 7a. The repeat allele sizes determined by lobSTR and ExpansionHunter are separated with a forward slash. Confidence intervals for allele sizes produced by ExpansionHunter are encoded similarly.

|  | AP < 0.9 | AP ≥ 0.9 |
| --- | --- | --- |
| WP < 0.9 | 305 | 35 |
| WP ≥ 0.9 | 139 | 1,145 |

**Supplementary Table 3.** Counts of putative anchored IRR with an absolute purity (AP) of at least 0.8 for the 182 ALS samples from cohort one identified by RP-PCR to have a pathogenic *C9orf72* repeat expansion. The read counts are stratified according to indicated WP and AP cutoffs.

**Supplementary Table 4.** ALS samples for which the pathogenic *C9orf72* repeat size call differed between the original RP-PCR and ExpansionHunter (EH). The table includes columns with counts of anchored IRRs and repeat sizes estimated from these counts. The remaining columns are the same as in Supplementary Table 2. See also Supplementary Figure 6.

**Supplementary Table 5.** Samples with wild type *C9orf72* repeats for which estimated sizes of longer alleles differed between fluorescent PCR and ExpansionHunter (EH). All columns are the same as in Supplementary Table 2. Consistent calls between EH and lobSTR where both disagree with the fluorescent PCR are not highlighted. Repeat sizes with a difference of one repeat between EH and and lobSTR are highlighted in yellow. Repeats that were originally not expanded, but were reclassified as expanded after re-evaluation of the RP-PCR and fluorescent PCR plots are highlighted in orange.

**Supplementary Table 6.** The read depth, Ti/Tv ratio, number of SNVs, indels and singletons is provided for all 3,001 samples used in the *C9orf72* repeat expansion study.

**Supplementary Table 7.** Phenotypes and sequencing metrics for 169 additional samples with eight additional validated repeat expansion sizes. When repeat size was not clear from the information supplied by Coriell, the NA was added to “Repeat Status” entry.

**Supplementary Table 8.** ExpansionHunter genotypes for all of the samples from Supplementary Table 7.


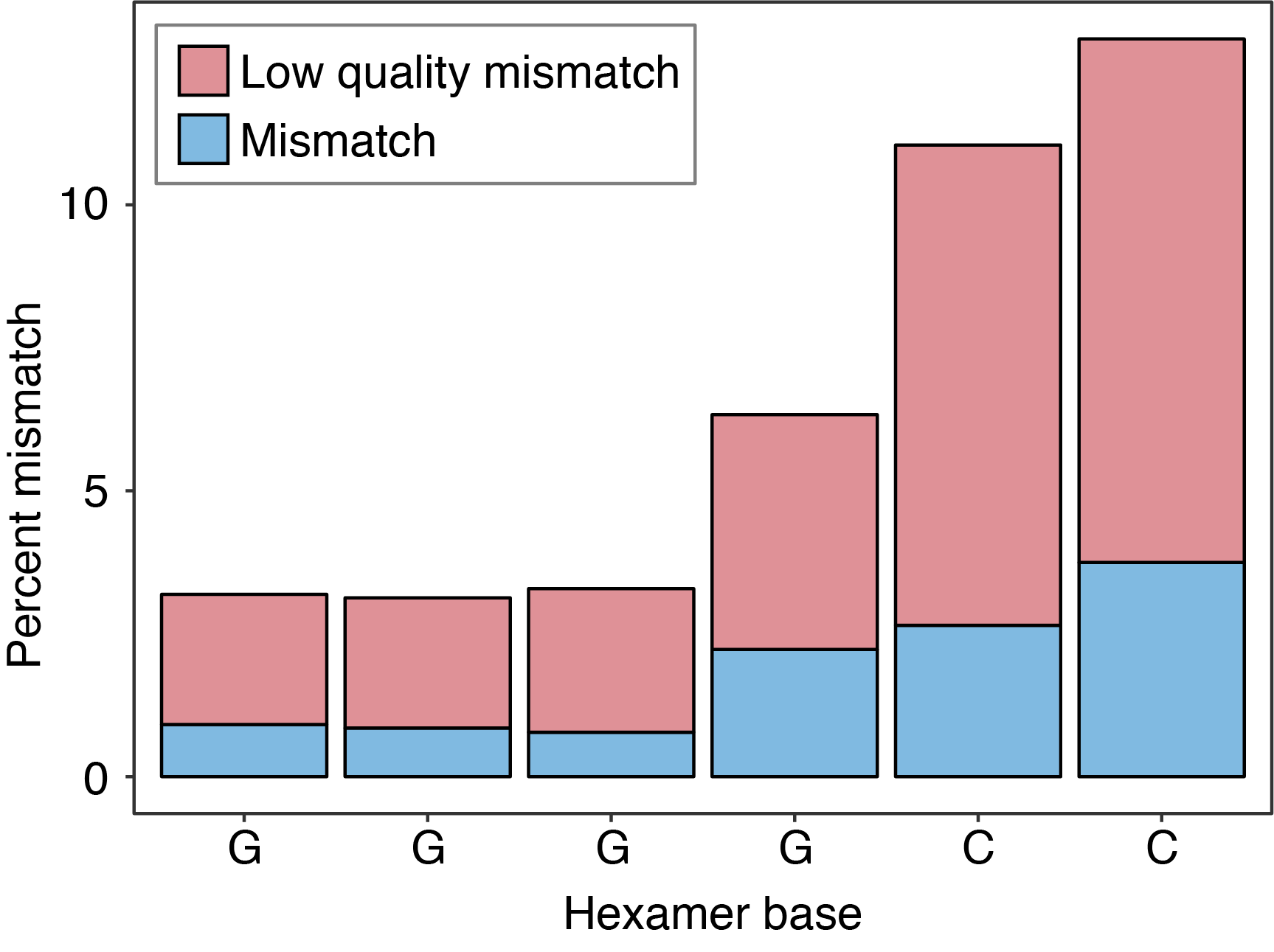


**Supplementary Figure 1.** Percent of mismatches between the expected base and the observed base in the *C9orf72* repeat motif for IRRs with an AP of at least 0.9. Low quality mismatches (<Q20) are red, high quality mismatches (≥Q20) are blue. The plot is based on reads aligned within 1Kb of the repeat region from the 182 *C9orf72* expanded samples according to RP-PCR in cohort one.


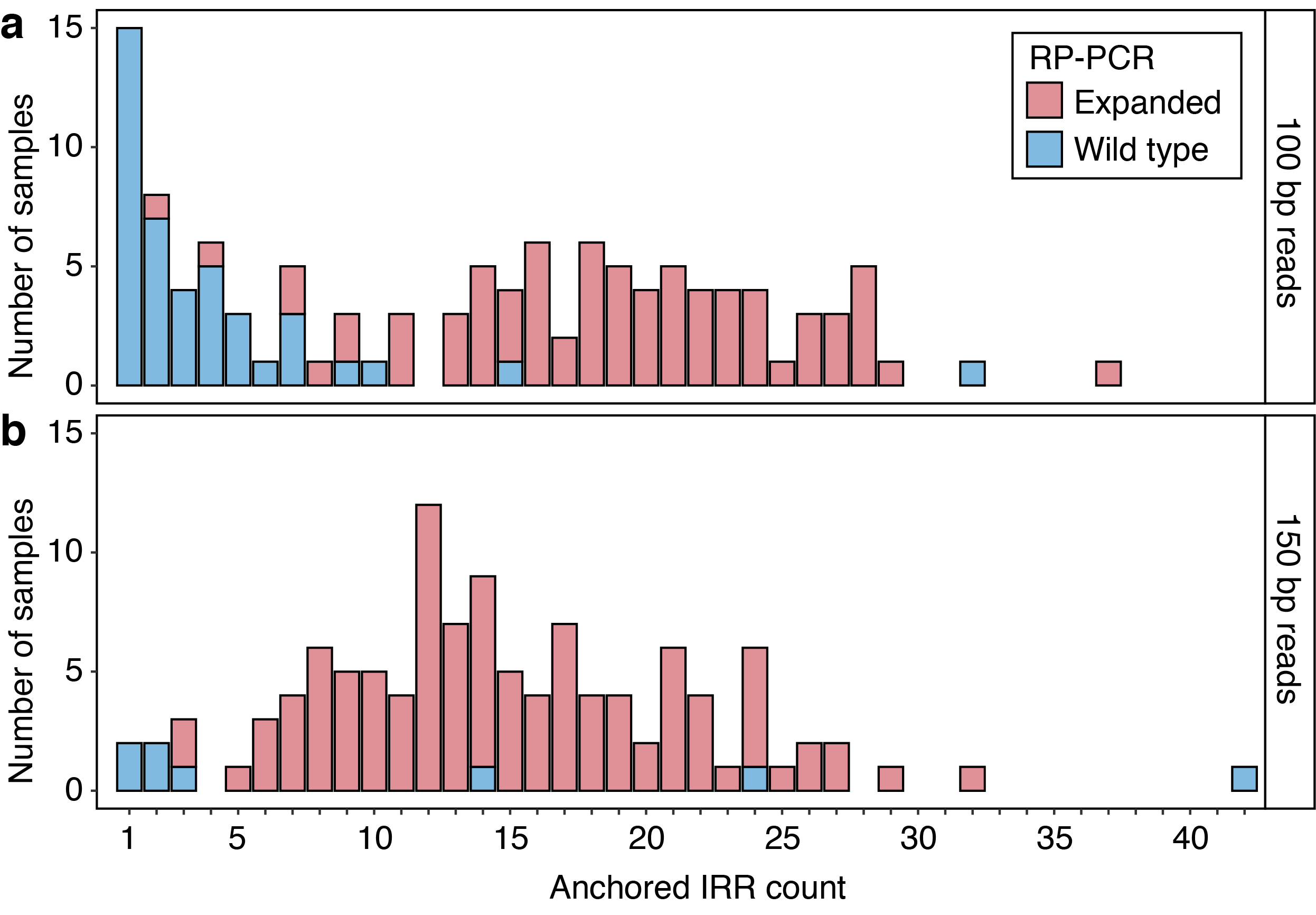


**Supplementary Figure 2.** The number of anchored IRRs for the (a) 117 samples sequenced with 2x100bp reads and (b) 114 samples sequenced with 2x150bp reads having at least one anchored IRR. Histograms are colored according to whether RP-PCR identified the *C9orf72* repeat as non-pathogenic (‘Wild type’) or pathogenic (‘Expanded’).

**
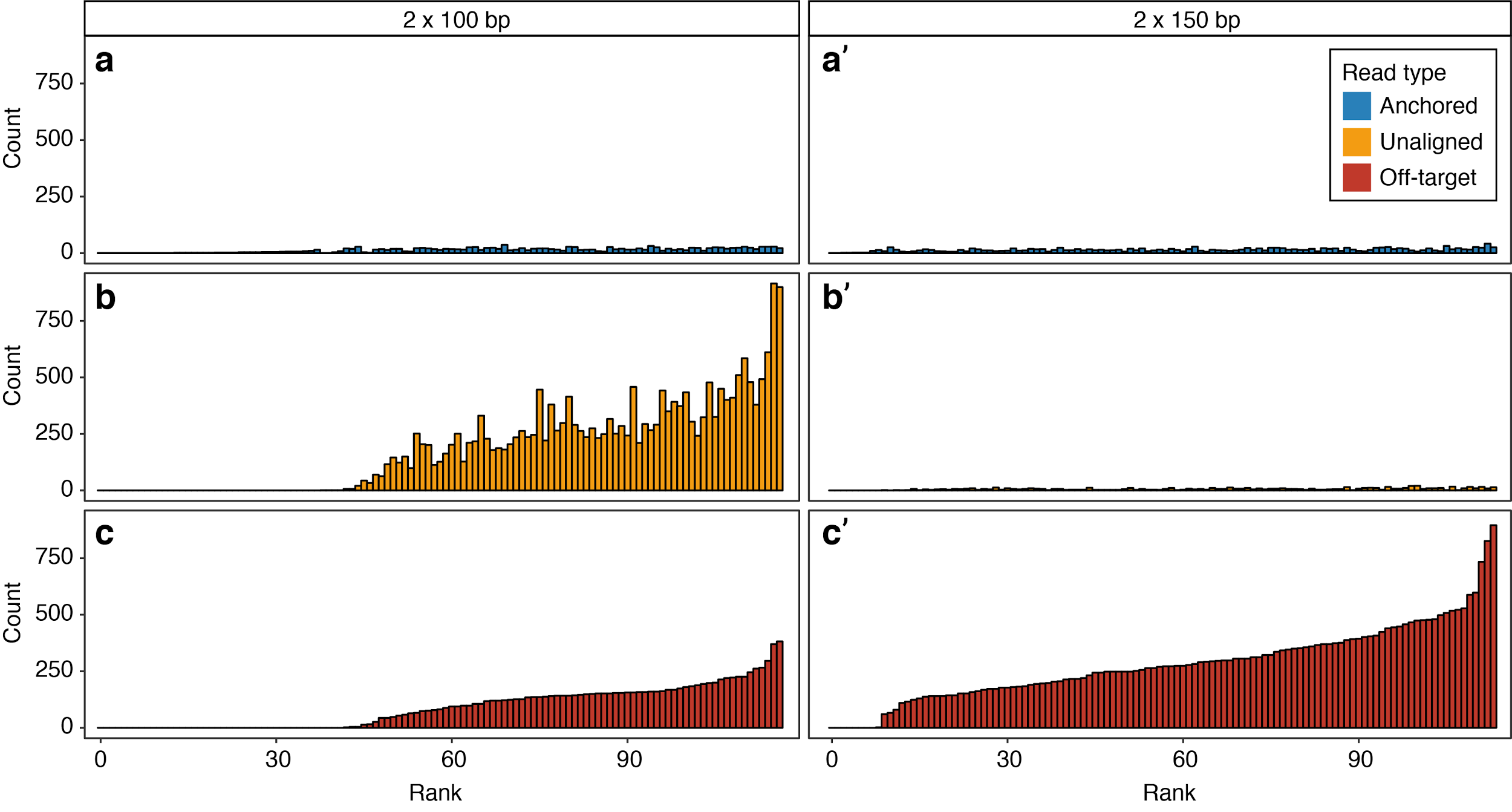
**

**Supplementary Figure 3.** Type and count of IRRs for the 117 2x100bp (a, b, c) and the 114 2x150bp (a’, b’, c’) samples of cohort one with at least one anchored IRR. The samples are ranked according to the total IRR count. For the 2x100bp samples more than half of the IRRs occurred in the unaligned reads, while for the 2x150bp samples the majority of the IRRs occurred in the off-target regions.


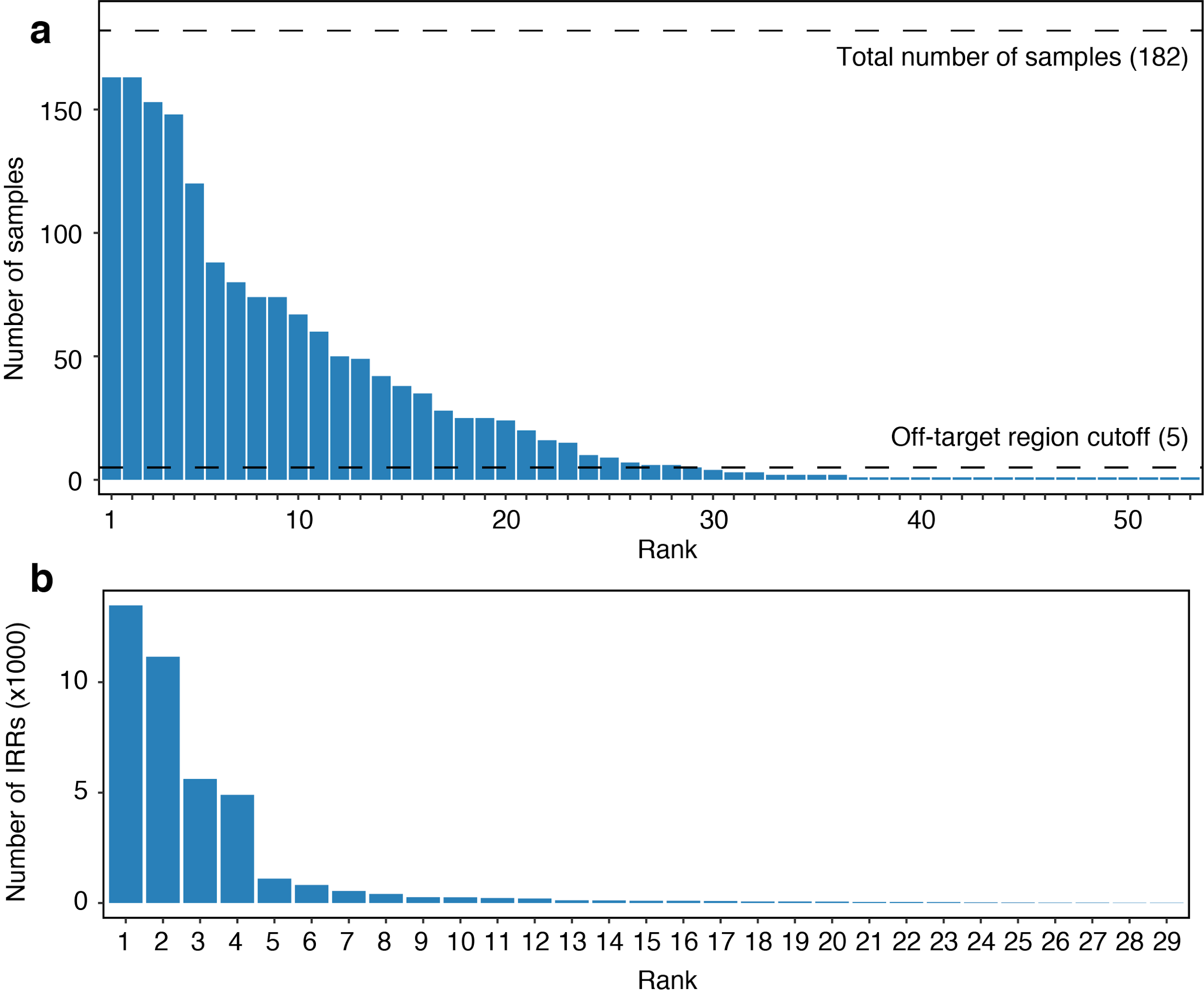


**Supplementary Figure 4.** (a) Ranking of genomic regions where at least two of the 182 *C9orf72* repeat expanded samples of cohort one contained an IRR pair with the GGGGCC repeat motif. Height of the bar gives the number of samples in which an IRR pair was observed at that genomic region. The 29 off-target regions used in this study are the genomic regions where an IRR pair was observed in at least 5 of the expanded samples (lower dashed line). (b) The 29 off-target regions used in this study ranked by the total number of IRR pairs identified at those locations. Over 99.9% of aligned IRR pairs were found in these 29 regions.


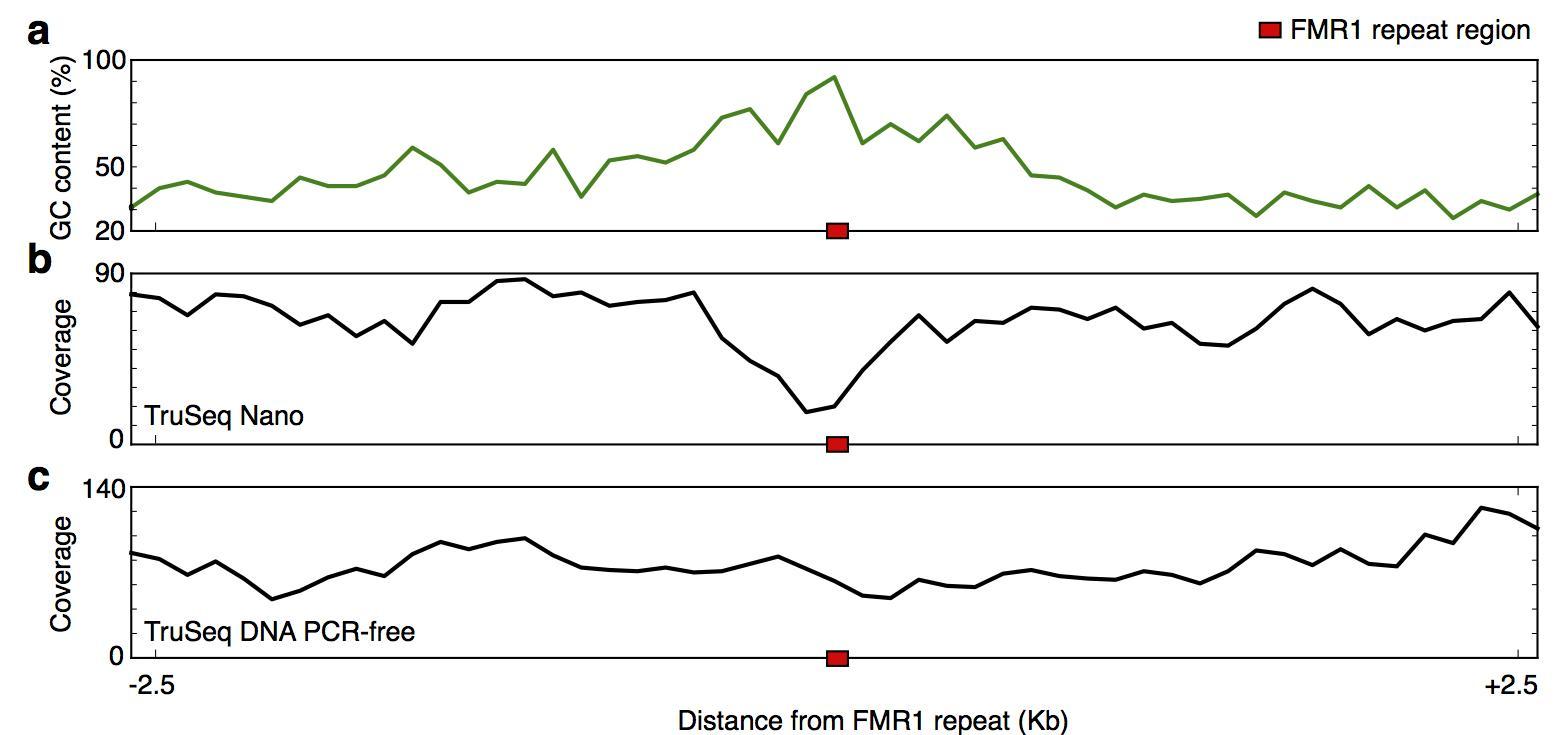


**Supplementary Figure 5.** GC content (top) and read coverage for a TruSeq Nano sample (middle) and a TruSeq DNA PCR-free sample (bottom) in a ~5kb region centered around the FMR1 repeat (red). The elevated GC content around the CGG repeat corresponds to lower depths in the TruSeq Nano sample. Both sequencing experiments use the NA12878 sample from the Platinum Genomes pedigree [(Eberle et al. 2017)](https://paperpile.com/c/M9MWlJ/DqIA).

**Supplementary Figure 6.** RP-PCR and fluorescent PCR plots of the twelve samples with a conflicting original RP-PCR and ExpansionHunter result (Supplementary Table 4). All fluorescent PCR plots had only one peak, indicating either a homozygous *C9orf72* repeat or the presence of a pathogenic repeat expansion. A sawtooth pattern in the RP-PCR plots indicates a pathogenic repeat expansion.


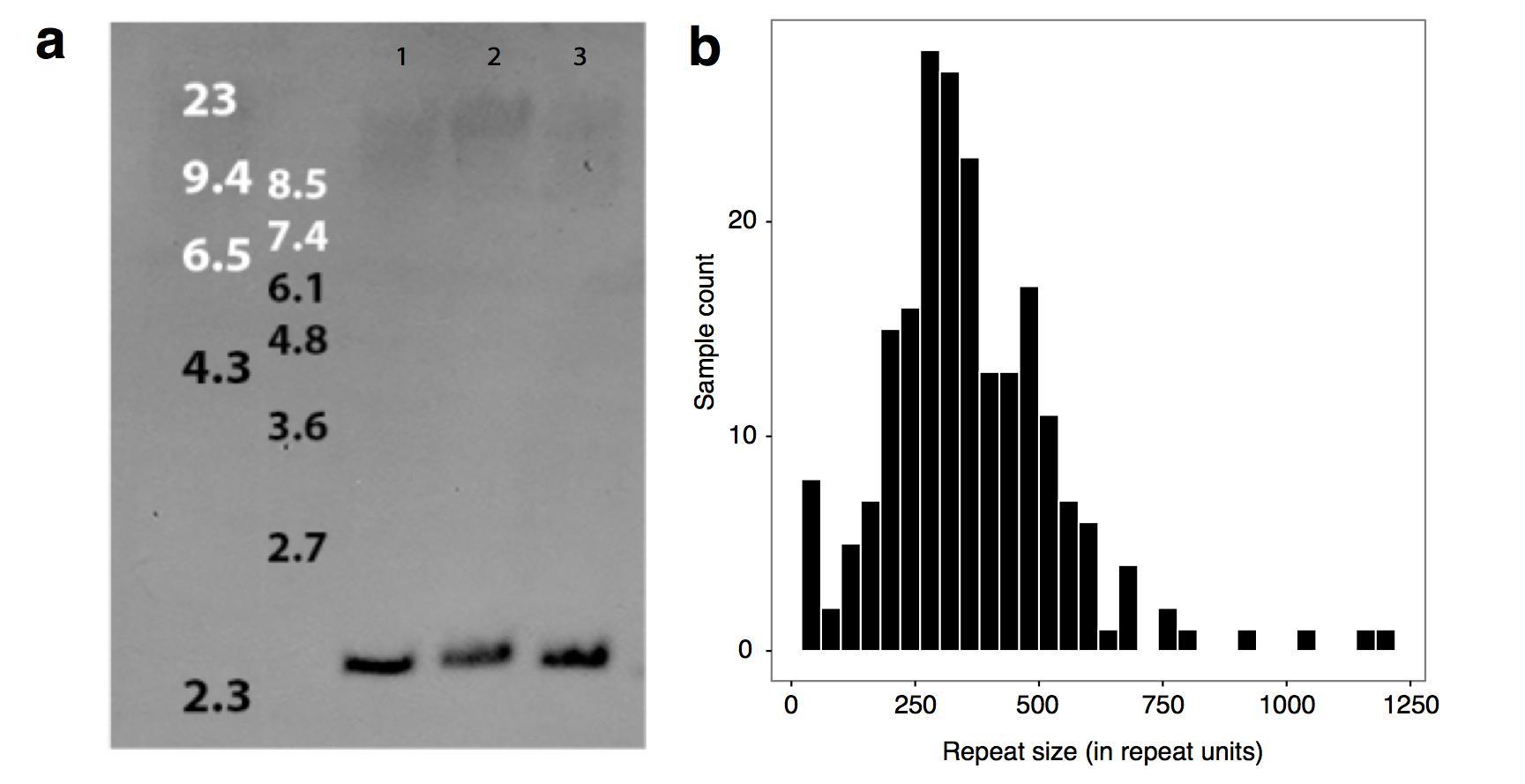


**Supplementary Figure 7.** (a) Southern blot of DNA that was isolated from fresh blood that had never been frozen from three ALS patients with a pathogenic *C9orf72* repeat expansion. (b) Histogram of repeat sizes for all samples from cohorts 1 and 2 flagged by ExpansionHunter as expanded. The repeat sizes estimated from the Southern blot (a) are listed in Supplementary Table 2.


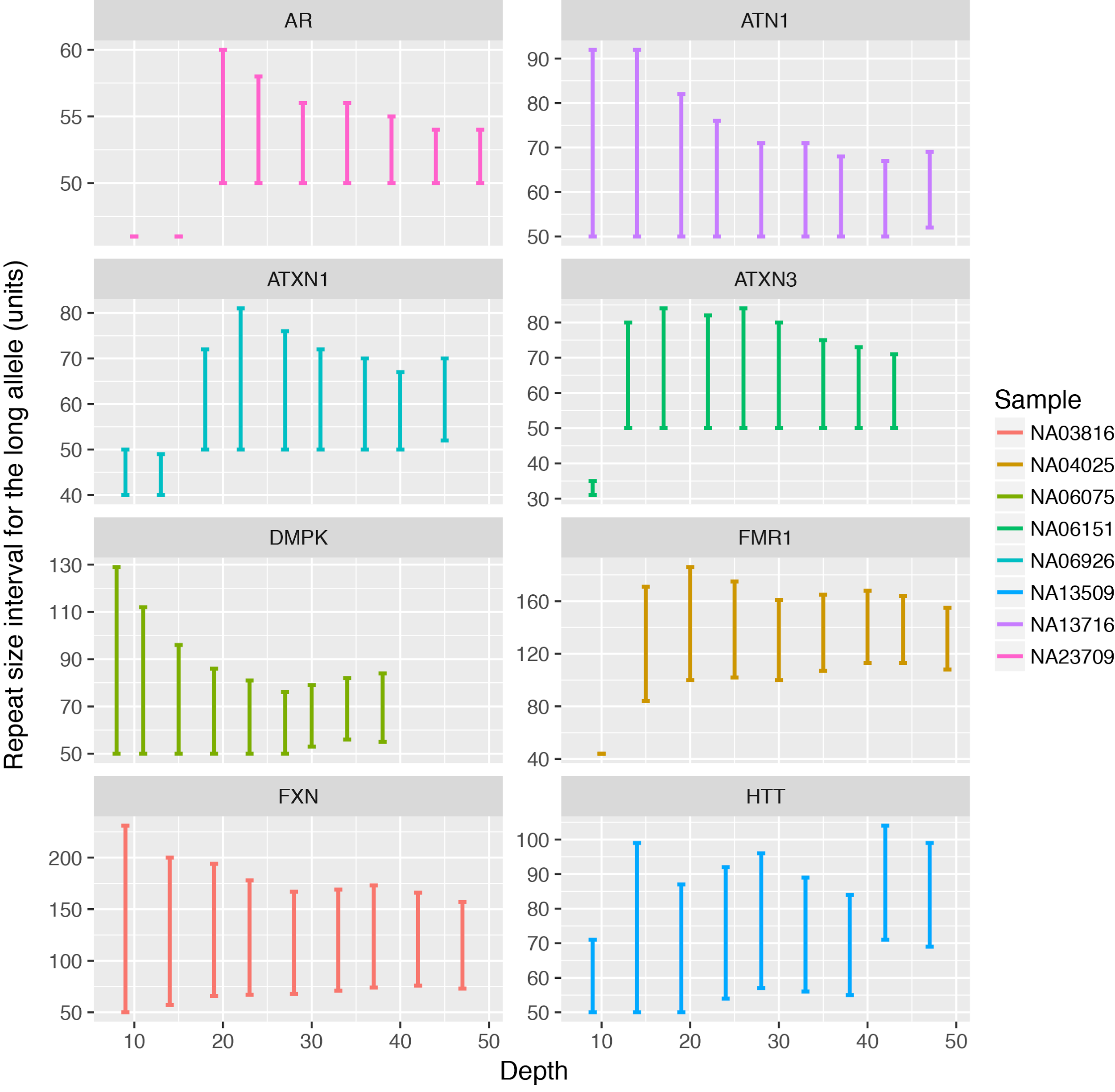


**Supplementary Figure 8.** Long allele repeat size confidence intervals estimated by Expansion Hunter for Coriell samples with expansions in 8 repeat regions when downsampling the data to lower depths. The size ranges in each plot correspond to (from left to right) libraries containing 20%, 30%, …, 100% of all reads contained in the original library. The x-axis corresponds to the mean read depths for each of the downsampled libraries.

## 2 Data generation

### 2.1 DNA isolation

Genomic DNA was extracted from whole blood using the Chemagic DNA Blood Kit (Chemagen) and stored at -80°C. To allow Southern blotting on DNA isolated from fresh whole blood, protocols were optimized to obtain DNA of optimal quality (and quantity) using the Gentra Puregene Blood Kit (Qiagen) [(van Blitterswijk et al. 2013)](https://paperpile.com/c/M9MWlJ/ICQU).

## 3 Supplementary Computational Methods

### 3.1 Absolute and Weighted Purity

To test whether a read fully consists of the repeat motif we created a set of simulated reads that were comprised of the repeat motif. Each simulated read was comprised of any of the possible motifs that would be observed both in the forward and reverse complement orientation, e.g. a read with a CAG repeat can consist of repetitions of either CAG, AGC, GCA in the forward orientation or CTG, TGC, GCT in the reverse orientation. Each read was compared with the simulated reads to identify the fraction of agreement. The absolute purity (AP) is the maximum fraction of matching bases observed for any of the comparisons between the read and the simulated reads. Reads with an AP of at least 0.9 (at least 90 consistent bases in a 100bp read and at least 135 consistent bases in a 150bp read) had on average ~6.7% mismatches (Supplementary Figure 1). The AP does not distinguish between mismatches due to the read overlapping some of the flanking sequence or due to low quality bases, for example caused by a high GC content in the repeat motif. However, ~72% of the mismatches occurred in low quality bases, defined as bases with a sequencing quality less than Q20 (Supplementary Figure 1).

To improve our ability to detect true IRRs with mismatches driven by lower base qualities in the repeat sequence we implemented a weighted purity (WP) score that penalizes mismatches at low quality bases less than at high quality bases. For the AP, each matching base has a score of 1, and each mismatch has a score of 0. For the WP, each matching base has a score of 1, each low quality mismatch has a score of 0.5 and each high quality mismatch has a score of -1. After normalization for the total read length the AP ranges from 0 to 1 and the WP ranges from -1 to 1. We chose a cutoff of 0.9 for our WP metric: a read with all low quality bases could pass this test with 20% mismatches (AP=0.8) but a read with all high quality bases may have at most 5% mismatches (AP=0.95). Of the total reads aligned within 1kb of the repeat region having an AP of at least 0.8, 1,145 were categorized as IRR using both the AP and WP while the WP recovered 139 reads that failed the AP and eliminated 35 reads that passed the AP cutoff. This is an increase of 8.8% in the number of WP IRRs compared to AP IRRs (Supplementary Table 3).

### 4 Supplementary Results

### 4.1 Analysis of *C9orf72* repeats by southern blot

Southern blotting was performed on 52 samples with more than 30 C9orf72 repeats (‘long’) and 16 samples with 15-30 repeats (‘wt’) according to RP-PCR (Supplementary Table 2). All 16 ‘wt’ samples had between 15-30 repeats according to the Southern blot and ExpansionHunter (Supplementary Table 2). One ‘long’ sample had an estimated repeat size number between 30-80 repeats according to the Southern blot, which was also predicted by ExpansionHunter (Supplementary Table 2). The other 51 ‘long’ samples revealed a DNA smear with more than 450 repeats on the Southern blot (Supplementary Table 2). That this was likely due to DNA degradation, was confirmed by extracting DNA directly after the donation of blood from three ALS patients (Supplementary information 2.1) and performing a Southern blot (Supplementary Figure 7a), because this revealed a much smaller DNA smear with a higher molecular weight. The DNA smear observed in the three fresh blood samples can reflect repeat instability (e.g., due to somatic heterogeneity). The average pathogenic C9orf72 repeat size estimated by ExpansionHunter was approximately 300 repeats (1,800 bp, Supplementary Figure 7b). Comparing the size estimates of the three ‘fresh-blood’ C9orf72 repeat expansions from the Southern blot with the ‘old-DNA’ WGS data from ExpansionHunter showed that the repeat sizes from the Southern blot were 3-5 times larger than those of ExpansionHunter (Supplementary Table 2). This discrepancy between the estimated repeat size range of ExpansionHunter and the ‘fresh-blood’ Southern blot, could be due to the fact that WGS was performed on the same (low quality) DNA on which assessing the pathogenic repeat size using Southern blot failed. Another concern is the high GC content of the C9orf72 repeat, which could cause a sequencing bias. It is important to use freshly isolated, non-frozen DNA when assessing the size of very long repeats, with Southern blot, WGS or any technique.

## 5 References

[DeJesus-Hernandez M, Mackenzie IR, Boeve BF, Boxer AL, Baker M, Rutherford NJ, Nicholson AM, Finch NA, Flynn H, Adamson J, et al. 2011. Expanded GGGGCC hexanucleotide repeat in noncoding region of C9ORF72 causes chromosome 9p-linked FTD and ALS. *Neuron* **72**: 245–256.](http://paperpile.com/b/M9MWlJ/sC23)

[Eberle MA, Fritzilas E, Krusche P, Källberg M, Moore BL, Bekritsky MA, Iqbal Z, Chuang H-Y, Humphray SJ, Halpern AL, et al. 2017. A reference data set of 5.4 million phased human variants validated by genetic inheritance from sequencing a three-generation 17-member pedigree. *Genome Res* **27**: 157–164.](http://paperpile.com/b/M9MWlJ/DqIA)

[Li R, Li Y, Fang X, Yang H, Wang J, Kristiansen K, Wang J. 2009. SNP detection for massively parallel whole-genome resequencing. *Genome Res* **19**: 1124–1132.](http://paperpile.com/b/M9MWlJ/M1Cx)

[Rice JA. 2007. *Mathematical Statistics and Data Analysis*.](http://paperpile.com/b/M9MWlJ/BpV0)

[van Blitterswijk M, DeJesus-Hernandez M, Niemantsverdriet E, Murray ME, Heckman MG, Diehl NN, Brown PH, Baker MC, Finch NA, Bauer PO, et al. 2013. Association between repeat sizes and clinical and pathological characteristics in carriers of C9ORF72 repeat expansions (Xpansize-72): a cross-sectional cohort study. *Lancet Neurol* **12**: 978–988.](http://paperpile.com/b/M9MWlJ/ICQU)
