# Supplementary material for: Detection of long repeat expansions from PCR-free whole-genome sequence data
Source: Genome Res. 2017 Nov;27(11):1895–903. doi: 10.1101/gr.225672.117 (PMC5668946; doi:10.1101/gr.225672.117)

a

Sample ID	Original RP-PCR	Final RP-PCR
LP6008186-DNAC09	Long	wt

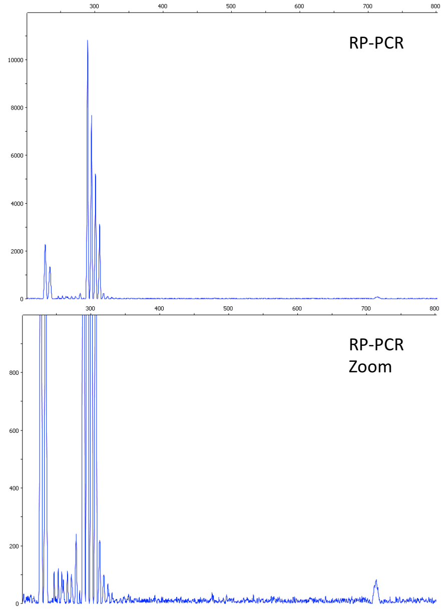

b

Sample ID	Original RP-PCR	Final RP-PCR
LP6005616-DNAA01	Long	Wt

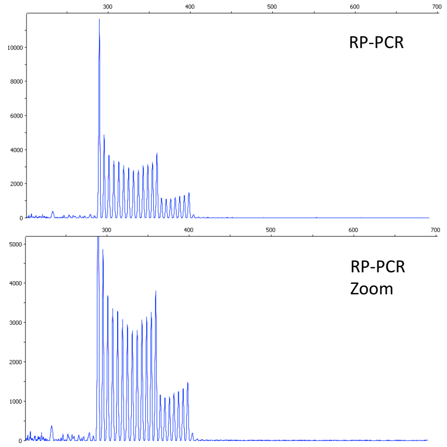

c

Sample ID	Original RP-PCR	Final RP-PCR
LP6008240-DNAE11	Long	Wt

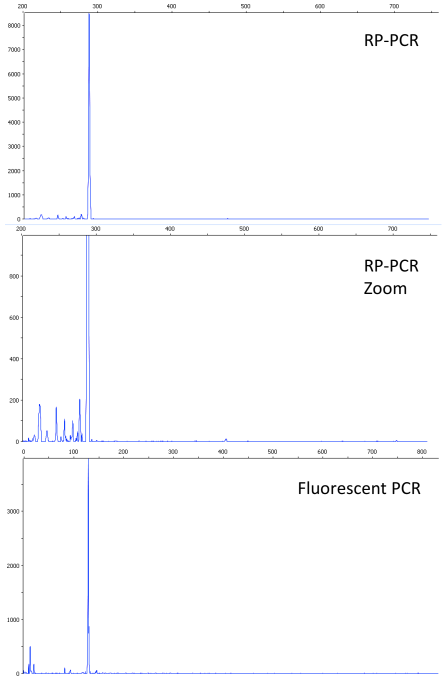

e

Sample ID	Original RP-PCR	Final RP-PCR
LP6008234-DNAB12	Wt	Long

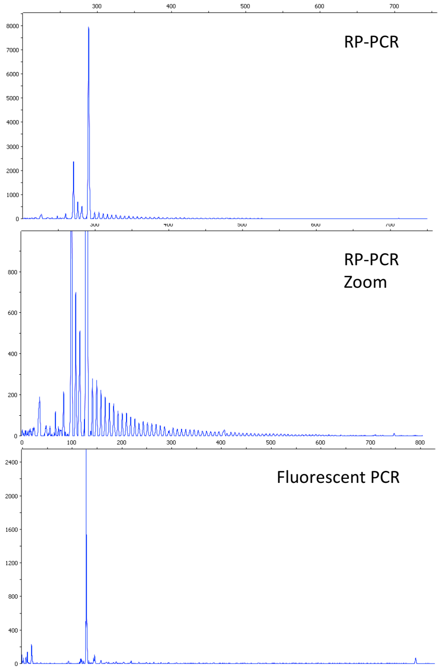

f

Sample ID	Original RP-PCR	Final RP-PCR
LP6008240-DNAC02	Wt	Long

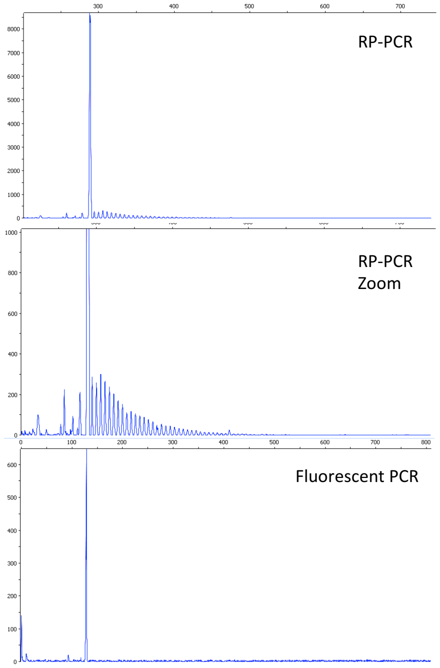

g

Sample ID	Original RP-PCR	Final RP-PCR
LP6008240-DNAA11	Wt	Long

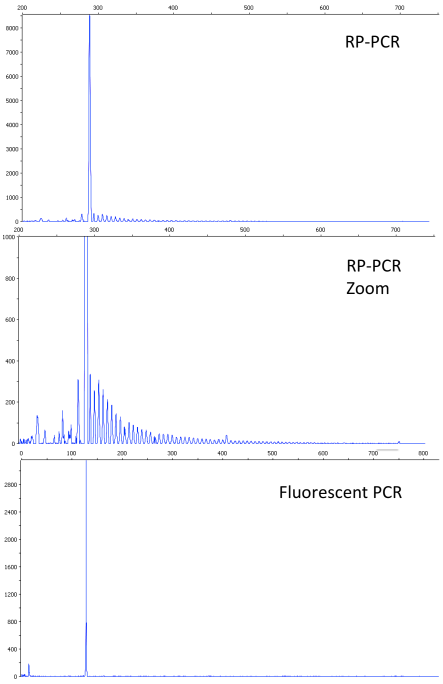

h

Sample ID	Original RP-PCR	Final RP-PCR
LP6008118-DNAG09	Wt	Long

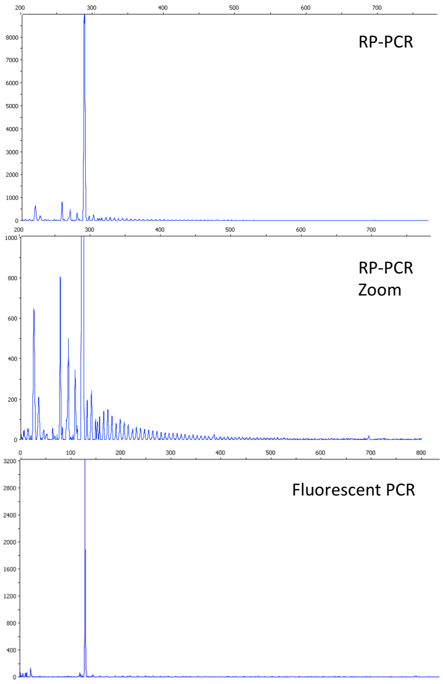

i

Sample ID	Original RP-PCR	Final RP-PCR
LP6005874-DNA_E11	Wt	Long

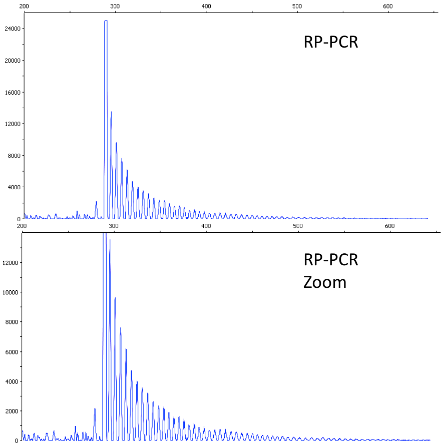

j

Sample ID	Original RP-PCR	Final RP-PCR
LP6005869-DNA_G04	Wt	Long

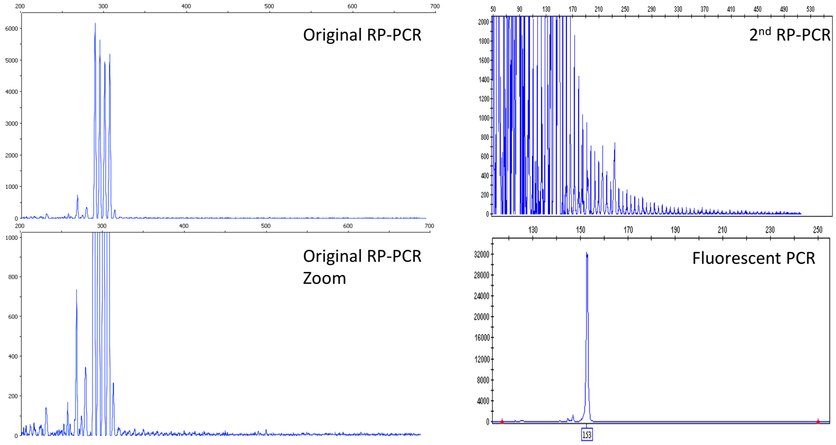

k

Sample ID	Original RP-PCR	Final RP-PCR
LP6008123-DNAG07	Wt	Long

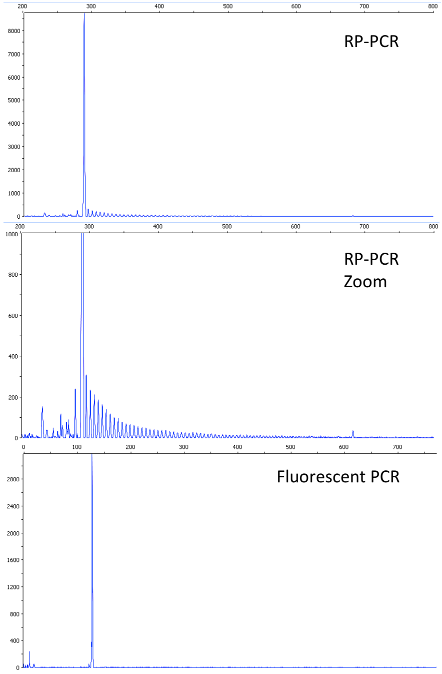

l

Sample ID	Original RP-PCR	Final RP-PCR
LP6008186-DNA_D04	Wt	Long

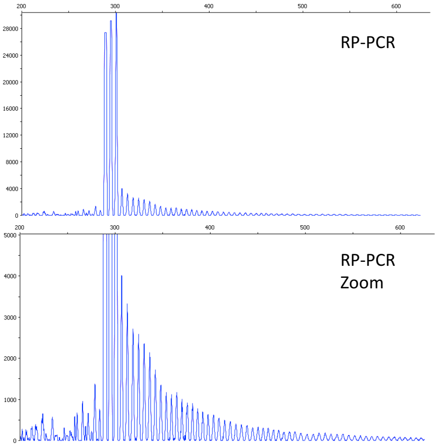

Supplement: Supplemental Material [file supp_gr.225672.117_Supplemental_Fig_6.pdf]
